# The evaluation of a virtual education system based on the DeLone and McLean model:  A path analysis

**DOI:** 10.12688/f1000research.12278.2

**Published:** 2017-09-25

**Authors:** Zohreh Mahmoodi, Sara Esmaelzadeh- Saeieh, Razieh Lotfi, Monir Baradaran Eftekhari, Mahnaz Akbari Kamrani, Zahra Mehdizadeh Tourzani, Katayoun Salehi

**Affiliations:** 1 Non-communicable Disease Research Center , Alborz University of Medical Sciences, Karaj, Iran; 2 Department of Midwifery School of Nursing and Midwifery, Alborz University of Medical Sciences, Karaj, Iran; 3 Undersecretary for Research & Technology, Ministry of Health and Medical Education , Tehran, Iran; 4 Social Determinant of Health Research Center, Alborz University of Medical Sciences, Karaj, Iran

**Keywords:** virtual education, evaluation, path analysis, model, DeLone and Mclean, education, system, e-learning

## Abstract

**Background**: The Internet has dramatically influenced the introduction of virtual education. Virtual education is a term that involves online education and e-learning. This study was conducted to evaluate a virtual education system based on the DeLone and McLean model.

**Methods**: This descriptive analytical study was conducted using the census method on all the students of the Nursing and Midwifery Department of Alborz University of Medical Sciences who had taken at least one online course in 2016-2017. Data were collected using a researcher-made questionnaire based on the DeLone and McLean model in six domains and then analyzed in SPSS-16 and LISREL-8.8 using the path analysis.

**Results**: The goodness of fit indices (GFI) of the model represent the desirability and good fit of the model, and the rational nature of the adjusted relationships between the variables based on a conceptual model (GFI = 0.98; RMSEA = 0.014).The results showed that system quality has the greatest impact on the net benefits of the system through both direct and indirect paths (β=0.52), service quality through the indirect path (β=0.03) and user satisfaction through the direct path (β=0.73).

**Conclusions**: According to the results, system quality has the greatest overall impact on the net benefits of the system, both directly and indirectly by affecting user satisfaction and the intention to use. System quality should therefore be further emphasized, to use these systems more efficiently.

## Introduction

Information and communication technology (ICT) has attracted the attention of scientific circles and organizations over recent years, with a focus on knowledge and rationality, and in order to efficiently employ the power of thinking, transfer repetitive tasks to machines and remove communication constraints. This attention means that virtual education or e-learning is now considered a major achievement of ICT, and has caused a number of scientific, research and cultural advances ^1^.

**Figure 1.**
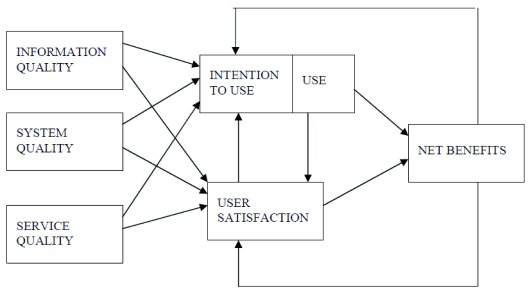
The DeLone and McLean updated theoretical model ^15^.

Clarke and Mayer define e-learning as presenting content through digital devices such as computers and cellphones, in order to improve learning ^2^. Using this method, learning takes place in a virtual environment in which learners interact with their peers, instructors and educational equipment in a non-traditional environment. In the virtual learning environment, the educational content is offered to the students through software, multimedia resources, the Internet and video conferencing, and the students communicate with their instructors, peers and other people or resources with the help of a computer, in order to carry out individual and group learning activities ^3, 4^. One of the benefits of this approach is the full-time and pervasive access to educational resources, reduced transportation time and costs for the students, the expansion of education for all at a much lower cost, the ease of access to multiple and diverse educational resources, the possibility of learning at any time and place and the possibility to learn from the instructor at any time and place ^5^. In spite of these benefits, implementing this form of education without analyzing whether the courses held have been adequately effective may lead to the failure of these courses as a means of education ^6^. One of the best and most-cited models used to evaluate the success of information systems is the DeLone and McLean model. This model measures the success of information systems by measuring six variables, including information quality, system quality, intention to use, user satisfaction, service quality and net benefits ( Figure 1). The success of a virtual medical education system is multidimensional and can be associated with system quality, information quality and service quality, which are the prerequisites of three other key structures, namely user satisfaction, intention to use and net benefits, which measure the effects of using the system after it is implemented ^7^. The evaluation of the courses held is one of the most important tasks of any organization and this need is twice as important in universities as they are considered a hub for research. The present study was conducted to evaluate the virtual education system implemented at the Nursing and Midwifery Department of Alborz University of Medical Sciences, based on the DeLone and McLean model using a path analysis.

## Methods

### Study design

This descriptive analytical study was conducted at the Nursing and Midwifery Department of Alborz University of Medical Sciences in Alborz, Iran, during the 2016–2017 academic years.

***Study participants.*** All the undergraduate and graduate nursing and midwifery students who had taken at least one online course (n=127) were enrolled in the study through census sampling.

### Data collection

Data were collected using a researcher-made questionnaire based on the DeLone and McLean model ( Supplementary File 1). The questionnaire was designed after a review of the literature and the relevant tools available, and was split into two sections:

(1) The socio-demographic details of the students (gender, age, field of study and marital status),

(2) System evaluation questions in six domains, including system quality (5 items), service quality (5 items), information quality (3 items), intention to use (3 items), user satisfaction (3 items) and net benefits (4 items), which were then scored based on a 5-point Likert scale (‘Totally disagree’ = 1 to ‘Totally agree’ = 5).

Once designed, the questionnaire was quantitatively and qualitatively validated using the face and content validity methods. For determining the face validity, the questionnaire was distributed among 15 students; for determining the content validity, it was distributed among ten faculty members in fields of nursing, reproductive health and management and they were asked to provide their feedback. All participants gave written consent for publication of potentially identifiable data. The reliability of the questionnaire was assessed using a test-retest with a two-week interval, and by measuring its Cronbach’s alpha coefficient.

### Procedures

The study began after obtaining the necessary permissions and a code of ethics from the university Ethics Committee (Abzums.Rec.1395.121). First, the eligible undergraduate and graduate nursing and midwifery students who had taken at least one online course in the 2016–2017 academic year were listed with the help of the Student Affairs Office. Arrangements were then made with this office in order to attend the online classes. At the final ten minutes of each class, the researcher went online and explained the objectives of the study, and obtained written consent from the students and provided them with the questionnaire to fill out.

The fit of a path analysis conceptual model ( Figure 1) was assessed in this study in order to determine the correlation between the variables used to evaluate the virtual education system. The path analysis method is a generalization of simple regression that shows not only the direct effects, but also the indirect effects and the effects of each of the variables on the dependent variables, and the results of such a study can be used to provide a rational interpretation of the observed relationships and correlations. The data obtained in this study were analyzed in LISREL version 8 software.

## Results

A total of 127 undergraduate and graduate students of the Nursing and Midwifery Department of Alborz University of Medical Sciences were recruited for this study. The mean age of the participants was 27.29 ± 1.5 years and the most frequent age group was 20–25 (49.6%). The majority of the participants were female (68.5%) and married (50.4%) ( Table 1).

**Table 1.** Frequency distribution of participant characteristics.

variable		f	%
age	20–25	63	49.7
	25–30	12	9.4
	30–35	24	18.9
	35–40	12	9.4
	>40	16	12.6
	Mean=27.2992±1.519		
sex	female	87	68.5
	male	40	31.5
Marriage statues	single	64	50.4
	marriage	61	48.0
	divorce	1	.8
	widow	1	.8

### Validity assessment

Based on the findings of the quantitative part of the face validity assessment, the impact score of all the items was between 2.4 and 3.3. Since the values were all more than 1.5, all the items remained in the questionnaire in this stage. In the content validity assessment stage, the content validity ratio (CVR) of all the items was between 0.63 and 0.8 and exceeded 0.62; the content validity index (CVI) of the items was also between 0.76 and 1 and exceeded 0.7. As a result, no items were removed from the questionnaire in this stage either. The Content Validity Index average (S-CVI/Ave) of the questionnaire was calculated as 96.37% (±0.65). The Cronbach’s alpha coefficient of the researcher-made questionnaire was 0.762, and the test-retest correlation coefficient with a two-week interval was 0.712.The questionnaire was thus approved for extensive research.

### Path analysis

To perform the path analysis, the correlation between the variables was investigated using the bivariate analysis. The net benefits of the system was the variable that had the highest correlation with user satisfaction (r = 0.81) and system quality (R = 0.72); ( Table 2).

**Table 2.** The correlation between the variables used in the evaluation of the education system.

	Quality of system	Quality of Information	Quality of service	Intention to use total	Satisfaction	Usefulness
Quality of system	1	.545**	.572**	.611**	.707**	.726**
Quality of Information		1	.404**	.389**	.468**	.524**
quality of service			1	.527**	.454**	.444**
Intention to use				1	.643**	.615**
satisfaction					1	.815**
usefulness						1

**Figure 2.**
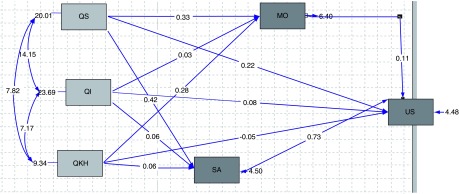
The full empirical path model for the effects of the variables used in the evaluation of the education system. QS: quality of system, QI: Quality of information, QKH: Quality of service, SA: satisfactory, MO: Intention to use, US: usefulness.

In the path analysis, the effects of variables including system quality, information quality, service quality, intention to use and user satisfaction were investigated for their usefulness ( Figure 2).

According to the results, the goodness of fit indices (GFI) of the model showed the desirability and good fit of the model and the rational nature of the adjusted relationships between the variables based on the conceptual model. As a result, the fitted model was not significantly different from the conceptual model ( Table 3).

**Table 3.** Goodness of fit indices for the model (N=127).

Model index	X ^2^	df	p	GFI	CFI	RMSEA
	0.05	1	0.82	0.98	0.95	0.014

According to the path diagram, user satisfaction (β = 0.73) was the variable that had the greatest impact on usefulness in the direct path and service quality (β = 0.03) the greatest impact in the indirect path. The only variable that had an impact through both the direct and indirect paths was system quality (β = 0.52).

According to the results, net benefits will be greater when the system is in a favorable condition in terms of these variables. Table 4 shows the direct, indirect and overall effects of the noted variables on usefulness.

**Table 4.** The path coefficients for the variables used in the evaluation of the education system.

Predictor variables	Effects			t-value	R ^2^	Errorvar
	Direct	Indirect	Total			
Quality of system	0.22	0.3066	0.5266	2.98	0.72	0.44
Quality of information	0.08	-	0.08	2.31		
Quality of service	-	0.0308	0.0308	-		
Intention to use	0.11		0.19	2.01		
satisfactory	0.73		0.73	5.9		

Raw data obtained from the questionnaire based on the DeLone and McLean model Gender, 1-female, 2-male; job, 1-student; marriage, 1-single, 2-married, 3-divorced, 4-widowed; education, 1-dipolma, 2-BS, 3-MS; subject, 1-medicine, 2-pharmacy, 3-dentistry, 4-nursing, 5-midwifery, 6-MS in consultation; QS1-5, question on quality of the system; QI1-5, question on quality of information; QH1-3, question on quality of service; M1-3, question on intention to use; S1-3, question on satisfaction; u1-4, question on usefulness. Click here for additional data file. Copyright: © 2017 Mahmoodi Z et al. 2017 Data associated with the article are available under the terms of the Creative Commons Zero "No rights reserved" data waiver (CC0 1.0 Public domain dedication).

## Discussion

Today, e-learning has become a growing trend and an important strategy for promoting education in all major countries of the world.

According to the findings of the study, system quality was the only variable that affected the benefits and usefulness of the virtual education system through both the direct and indirect paths. According to the DeLone and McLean model, system quality refers to aspects of the information system itself, such as data reception speed, easy access, system authenticity, responsiveness, flexibility and user-friendliness ^8– 11^. These findings are consistent with the results obtained by Pérez-Mira (2010) and Boroufar *et al*. (2014), who found that system quality has a positive impact on the benefits and usefulness of the system. According to their findings, system quality can be both encouraging and discouraging for the use of the system. They further argued that creating a quality e-learning system by increasing learner satisfaction has a positive effect on usefulness and benefits ^12, 13^. Chiu *et al*. (2005) also found that quality and motivation are two key factors that affect user satisfaction and continued use of virtual education. Machado *et al*. (2014) found that increased use has a direct relationship with increased system quality, and argued that people are encouraged to use virtual education systems that are user-friendly ^11^.

Of the variables exerting their effects only through one path, service quality had the greatest impact on benefits and usefulness by indirectly affecting the intention to use. According to the DeLone and McLean model, service quality is essential for implementing information systems and depends on the performance of the service providers, which require training in sales and customer support. In the present study, service quality was found to affect the net benefits and usefulness of the system by indirectly affecting learner’s intention. This finding is consistent with the results of a study by Boroufar *et al*. (2014), who found that service quality indirectly affects the net benefits and usefulness of a system by affecting user satisfaction. Kazemi and Nematollahi (2014) also found that if web-based learning meets the needs of the learners, they will be motivated and satisfied, and this motivation and satisfaction will then make the learners continue using the system ^14^.

Of the variables that directly affect the benefits of using the system, user satisfaction was the most effective. According to the DeLone and McLean model, user satisfaction refers to the response of the individual who uses the information system, such as overall satisfaction, enjoyable experiences and overall success. Chang *et al*. (2011) also found that the learners’ level of satisfaction has the greatest direct effect on net benefits (7 The term ‘net benefits’ refers to the main benefits achieved by user’s increased use and satisfaction ^8– 11^. The satisfaction of the users of a system plays a large role in the success and continued use of a system ^8^. In the present study, student satisfaction was affected by system quality and service quality. These results are consistent with the results obtained by Bauk *et al*., who also found that user satisfaction is an effective measure of the contradictions between the expectations before and the perceived performance after receiving the education. User satisfaction is affected by system quality while it affects the net benefits of the system. Pérez-Mira B (2010), however, found different results and stated that despite there being relationship between user satisfaction and net benefits, there are no significant relationships between these two variables when entered into a path analysis model, which could be due to the lack of sufficient variation in measuring the satisfaction variable ^13^.

### Limitations

A major limitation of this study lies in the lack of variability with regard to the courses offered through the virtual education system, which were limited to the Nursing and Midwifery Department of Alborz University of Medical Sciences.

## Conclusions

According to the results, system quality has the greatest overall impact on net benefits through both the direct and indirect paths by affecting user satisfaction and intention to use. The continued use of virtual education systems and the more efficient use of these systems therefore requires special attention to system quality, which involves speed of data transfer, ease of access, system authenticity, responsiveness, flexibility and user-friendliness.

## Data availability

The data referenced by this article are under copyright with the following copyright statement: Copyright: © 2017 Mahmoodi Z et al.

Data associated with the article are available under the terms of the Creative Commons Zero "No rights reserved" data waiver (CC0 1.0 Public domain dedication). 

**Dataset 1: Raw data obtained from the questionnaire based on the DeLone and McLean model.** Gender, 1-female, 2-male; job, 1-student; marriage, 1-single, 2-married, 3-divorced, 4-widowed; education, 1-dipolma, 2-BS, 3-MS; subject, 1-medicine, 2-pharmacy, 3-dentistry, 4-nursing, 5-midwifery, 6-MS in consultation; QS1-5, question on quality of the system; QI1-5, question on quality of information; QH1-3, question on quality of service; M1-3, question on intention to use; S1-3, question on satisfaction; u1-4, question on usefulness. doi, 10.5256/f1000research.12278.d175011 ^16^

## Supplementary material

Supplementary File 1: Questionnaire based on the Delone and McLean model.
