# Supplementary material for: The evaluation of a virtual education system based on the DeLone and McLean model: A path analysis
Source: F1000Res. 2017 Sep 25;6:1631. Originally published 2017 Sep 4. [Version 2] doi: 10.12688/f1000research.12278.2 (PMC5599998; doi:10.12688/f1000research.12278.2)
Supplement: Supplementary file 2 [file f1000research-6-13811-s0001.tgz › ef18fddd-5224-4cca-9b35-b588b027e330.docx]

Questionnaire based on the DeLone and McLean model

1= I totally disagree, 2=disagree , 3= somewhat agree, 4= agree 5= I totally agree

| Quality of system | speed | The e-Learning system features high-speed information reception | 1 | 2 | 3 | 4 | 5 |
| --- | --- | --- | --- | --- | --- | --- | --- |
|  | ease of use | The e-Learning system is easy to learn and takes little effort to master |  |  |  |  |  |
|  | Reliability | The e-Learning system seldom delays training due to a software or hardware crash or error |  |  |  |  |  |
|  | Usability | The e-learning system provides services for facilitating day-to-day education |  |  |  |  |  |
|  | Being accountable | The e-learning system features a quick response. Therefore, waiting is not long and does not prevent learning activities. |  |  |  |  |  |
| Quality of Information | accuracy | The e-learning system provides accurate educational information |  |  |  |  |  |
|  | Sufficiency | The e-learning system provides complete and relevant information. |  |  |  |  |  |
|  | On Time | The e-learning system provides timely and required training information. |  |  |  |  |  |
|  | Security | The e-learning system retains the student's personal information |  |  |  |  |  |
|  | Understandable | The e-learning system provides understandable information |  |  |  |  |  |
| Quality of service | Being accountable | Personnel respond quickly to student problems and requests related to the e-learning system |  |  |  |  |  |
|  | assurance | Personnel clearly understand the needs of students when they use the e-learning system |  |  |  |  |  |
|  | Support | The E-Learning Staff has an immediate and online support service for students |  |  |  |  |  |
| Intention to use | Availability | An e-learning system that is easily accessible |  |  |  |  |  |
|  | Necessity | Students consider the use of e-learning as essential |  |  |  |  |  |
|  | References | The resources used in the e-learning system are suitable for learning |  |  |  |  |  |
| satisfaction | usefulness | Students feel that the e-learning system is useful |  |  |  |  |  |
|  | Overall satisfaction | In general, students are satisfied with the use of the e-learning system |  |  |  |  |  |
|  | Enjoyable experiences | Students have enjoyable experiences when using an e-learning system. |  |  |  |  |  |
| usefulness | Incremental Productivity | Using the e-learning system increases student productivity |  |  |  |  |  |
|  | Incremental Effectiveness | Using the e-learning system increases the effectiveness of student education |  |  |  |  |  |
|  | Save time | Using e-learning systems, less time is spent on teaching students |  |  |  |  |  |
|  | ability | Using the e-learning system, students' ability to learn increases |  |  |  |  |  |
