# Supplementary material for: The evaluation of a virtual education system based on the DeLone and McLean model:  A path analysis
Source: F1000Res. 2017 Sep 25;6:1631. Originally published 2017 Sep 4. [Version 2] doi: 10.12688/f1000research.12278.2 (PMC5599998; doi:10.12688/f1000research.12278.2)
Supplement: Peer review history

# Peer review: Referee response for version 1

**DOI:** 10.5256/f1000research.13292.r25649

**Published:** 2017-09-20

**Authors:** Laila Amini

**Affiliations:** 1 Department of Midwifery and Reproductive Health, Faculty of Medical Sciences, Tarbiat Modares University, Tehran, Iran

- The total percentage of age in table is not 100% please correct
- Please change subgroups of sex in table 1 to male and female  or  women and men
- And marital statues change to married, divorced,...
- Introduction and discussion parts are not supported with enough documents and it is better researchers review more references in their article

I have read this submission. I believe that I have an appropriate level of expertise to confirm that it is of an acceptable scientific standard.

# Response

**Published:** 2017-09-20

**Authors:** Zohreh Mahmoodi

**Affiliations:** Assistant Professor, Non-communicable Disease Research Center ,Alborz University of Medical Sciences, Karaj, Iran, Iran

Dear Editor thank you for your decision.

 

- The total percentage of age in table is not 100% please correct

Answer: It was corrected

- Please change subgroups of sex in table 1 to male and female  or  women and men

Answer: It was corrected

- Introduction and discussion parts are not supported with enough documents and it is better researchers review more references in their article

Answer: I reviewed it again

# Peer review: Referee response for version 1

**DOI:** 10.5256/f1000research.13292.r25972

**Published:** 2017-09-13

**Authors:** Parisa Shojaee

**Affiliations:** 1 Social Determinants Of Health Research Center, University of Social Welfare and Rehabilitation Sciences, Tehran, Iran

"...Student Affairs Office. Arrangements were then made with this office in order to attend the online classes. **At the final ten minutes of each class**, the researcher went online and explained the objectives of the study, and obtained written consent from the students and provided them with the questionnaire to fill out."

 In my opinion, the completion time of the questionnaire was not suitable due to the stress of the exam. More exact details should be explained about the path analysis for readers of this paper.

I have read this submission. I believe that I have an appropriate level of expertise to confirm that it is of an acceptable scientific standard.

# Response

**Published:** 2017-09-14

**Authors:** Zohreh Mahmoodi

**Affiliations:** Assistant Professor, Non-communicable Disease Research Center ,Alborz University of Medical Sciences, Karaj, Iran, Iran

Dear Editor Thanks for your decision and your reviewer comments.

1-In my opinion, the completion time of the questionnaire was not suitable due to the stress of the exam. More exact details should be explained about the path analysis for readers of this paper.

Answer:The questionnaire was complete at the end of each class so the sentence  ' **At the final ten minutes of each class' **means at the end of class not the exam time.

Regards.

# Peer review: Referee response for version 1

**DOI:** 10.5256/f1000research.13292.r25652

**Published:** 2017-09-07

**Authors:** Mahboubeh Ahmadi

**Affiliations:** 1 Department of Midwifery and Reproductive Health, School of Nursing and Midwifery, Shahid Beheshti University of Medical Sciences, Tehran, Iran

Accurate design and complete execution, key points are the main strength of this study.

 The research topic is new and very interesting. However, it requires to answer some question.

1. Why was this project only in the Nursing and Midwifery faculty?
2. Why did they use this model for evaluation?

I have read this submission. I believe that I have an appropriate level of expertise to confirm that it is of an acceptable scientific standard.

# Response

**Published:** 2017-09-07

**Authors:** Zohreh Mahmoodi

**Affiliations:** Assistant Professor, Non-communicable Disease Research Center ,Alborz University of Medical Sciences, Karaj, Iran, Iran

Dear Editor Thanks for your decision and your reviewer comments.

Comments for the Author:

*1- Why was this project only in the Nursing and Midwifery faculty?*

Answer: We did it in Nursing and Midwifery Department only because, the courses offered through the virtual education system, were limited to this Department and this subject was not presented in other department of Alborz University of Medical Sciences. I wrote it in limitation part.

*2- Why did they use this model for evaluation?*

Answer: One of the best and most-cited models used to evaluate the success of information systems is the DeLone and McLean model. This model measures the success of information systems by measuring six variables, including information quality, system quality, and intention to use, user satisfaction, service quality and net benefits
